# Deep learning and object detection methods for scoring cell types within the human buccal cell micronucleus and cytome assays for human biomonitoring

**DOI:** 10.1093/mutage/geaf026

**Published:** 2025-11-14

**Authors:** Eloise Smith, Jade Wagman, Claire Barnes, Paul Rees, George Johnson

**Affiliations:** Department of Biomedical Engineering, Faculty of Science and Engineering, Bay Campus, Swansea University, Fabian Way, Crymlyn Burrows, Skewen, SA18EN Swansea, United Kingdom; Faculty of Medicine, Singleton Campus, Swansea University, SA2 8PP Swansea, United Kingdom; Department of Biomedical Engineering, Faculty of Science and Engineering, Bay Campus, Swansea University, Fabian Way, Crymlyn Burrows, Skewen, SA18EN Swansea, United Kingdom; Department of Biomedical Engineering, Faculty of Science and Engineering, Bay Campus, Swansea University, Fabian Way, Crymlyn Burrows, Skewen, SA18EN Swansea, United Kingdom; Imaging Platform, Broad Institute of MIT and Harvard, Richard N. Merkin Building, 415 Main Street, Cambridge, MA 02142, United States; Faculty of Medicine, Singleton Campus, Swansea University, SA2 8PP Swansea, United Kingdom

**Keywords:** deep learning, object detection, micronuclei, buccal cell, human biomonitoring, cytome assay

## Abstract

Micronuclei (MN) are critical biomarkers for pathological conditions, yet their manual scoring is inherently laborious and prone to significant interobserver variability, limiting the reliability and scalability of genotoxicity assessments. Recent advancements in deep learning and computer vision have revolutionized automated MN detection in various assay samples, enhancing accuracy and efficiency and reducing human bias. While these artificial intelligence (AI)-powered techniques have been demonstrated in *in vitro* genotoxicity testing, their application to the minimally invasive buccal micronucleus cytome (BMCyt) assay for human biomonitoring remains largely unexplored. The BMCyt assay, invaluable for assessing genotoxic damage in environmentally exposed populations, presents unique challenges, including sample variability, confounding factors, and the complexity of scoring multiple cytogenetic endpoints. This review covers the evolution of AI-based MN detection, analysing key methodologies and advancements. It highlights the untapped potential of integrating AI into the BMCyt assay to overcome current analytical limitations, improve reproducibility, increase throughput, and eliminate observer bias. By facilitating more robust and scalable genomic damage monitoring, AI integration will significantly enhance the utility of the BMCyt assay in large-scale epidemiological studies and human biomonitoring.

## Introduction

Micronuclei (MN) are small, extranuclear bodies containing DNA, frequently observed in pathological conditions such as cancer. These structures serve as excellent biomarkers for disease and DNA damage caused by chemical or radiative exposure [1]. However, the manual scoring of MN is an arduous task, prone to significant person-to-person variation [2–4]. Even when the same slides are scored by different scientists, substantial discrepancies often arise, highlighting a critical need for automated scoring methods (e.g. flow cytometry, image analysis, and artificial intelligence [AI]) to reduce the inherent variability of manual assessments [5].

Given these limitations, researchers have increasingly explored automated and AI-based approaches to enhance the accuracy, efficiency, and reproducibility of micronucleus (MN) detection. Many of these approaches have been adopted from current advancements in the field of computer vision, an area of AI that enables computers to process visual information and aims to replicate human visual abilities. Recent advances in these techniques along with deep learning and machine learning have enabled automated image-based classification and segmentation of MN in assay samples, truly revolutionizing the field [4, 6, 7]. Specifically, AI-powered techniques developed for computer vision applications, such as classification and object detection neural networks [8], have significantly improved the identification of MN across various assay types [9, 10]. These methods not only reduce human bias but also enhance sensitivity in detecting small and complex features within cells [11–13].

**Figure 1:**
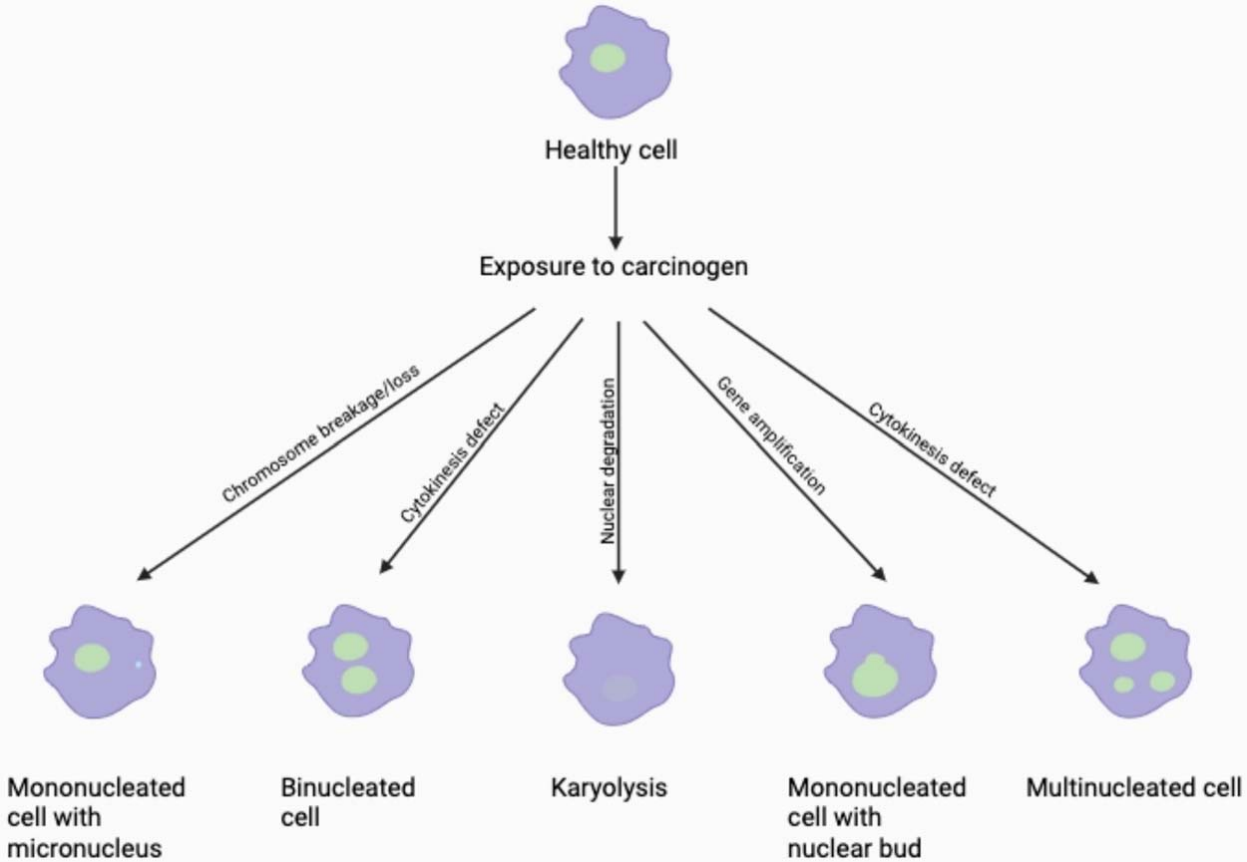
Key different buccal cell types formed through chromosome damage. For more detailed information about buccal cell types scored in the buccal cell MN assay, refer to the papers by Thomas *et al.* [14] and Bolognesi *et al.* [15]. Created in Biorender.com.

While the MN assay has broad applicability in genotoxicity assessment, the buccal micronucleus cytome (BMCyt) assay offers a distinct, minimally invasive approach particularly suited for human biomonitoring [14, 15]. Unlike the *in vitro* assay typically performed on cultured lymphocytes, the BMCyt assay utilizes exfoliated buccal cells, providing a direct reflection of genotoxic damage and cytotoxicity in a rapidly dividing epithelial tissue that is in constant contact with environmental agents [14, 15]. This noninvasive collection method makes it highly appealing for large-scale epidemiological studies, especially in vulnerable populations such as children or those undergoing long-term exposure monitoring [16]. However, the BMCyt assay presents its own set of challenges, including variability due to factors like hydration, oral hygiene, and inflammation; the presence of confounding factors such as bacteria and food debris; and the complexity of scoring multiple cytogenetic endpoints (like binucleated cells, karyorrhectic cells, condensed chromatin cells, and pyknotic cells) in addition to micronuclei (Fig. 1), which requires extensive training and standardized protocols [14, 15].

Currently machine learning and AI methods have not been employed to score the buccal cell MN assay, although no significant challenges stand in the way of the same approaches. This review aims to examine the evolution of AI-based MN detection, analysing key studies, methodologies, and advancements in the field. It will also explore challenges and future directions for AI integration in genotoxicity testing and human biomonitoring.

## Micronucleus assay in biomonitoring

The MN assay has also been adapted for use in human biomonitoring with buccal epithelial cells as a population-accessible, noninvasive substitute to cytogenetic tests based on blood. It is especially applicable to estimate genotoxic impacts in environmentally or occupationally exposed populations such as children and other vulnerable groups [16]. Not only does the BMCyt assay contribute to the detection of MN, but also it covers other endpoints such as nuclear buds, karyorrhexis, karyolysis, and pyknosis that provide information on genotoxicity and cytotoxicity [14]. These strengths render it a broader indicator for evaluating cell stress responses and health hazards. Given that biomonitoring studies mirror real-world exposure and heterogeneous populations, strict standardization of sampling, staining, and scoring procedures to ensure data validity is necessary. Large-scale multidisciplinary collaborative studies like the HUman MicroNucleus (HUMN) project have been responsible for overcoming these challenges [17]. By enabling international pooling of data, methodological standardization, and reference range establishment, the HUMN project has enabled the buccal MN assay as an internationally reproducible and validated biomarker of early biological effect and exposure [14]. Its use in biomonitoring not only enables large-scale monitoring of health but also supports epidemiological studies linking genotoxic exposure to long-term disease risk [13, 18].

The use of AI in buccal cell MN tests has the potential to enhance biomonitoring by transcending the limitations of conventional analysis [13, 18]. Reproducible and effective MN and other nuclear anomaly scoring is required in population-scale studies to yield viable exposure estimates. AI-based image analysis software, particularly deep learning–based AI software, can be applied to automate MN and cytotoxic marker detection and classification in exfoliated buccal cells, increasing throughput and avoiding observer bias. This is particularly valuable in biomonitoring, where interlaboratory standardization and high-quality datasets are required. By application of automated scoring under standardized protocols, AI can facilitate more stable and scalable genomic damage monitoring across human populations.

## Traditional micronucleus detection methods

MN detection has been traditionally performed through the visual evaluation of stained cell samples using microscopy. Examples of these common staining techniques include Giemsa, acridine orange, and 4′,6-diamidino-2-phenylindole (DAPI), which highlight nuclear material to enable the identification of MN. Guidelines such as those produced by the Organisation for Economic Co-operation and Development (OECD) for the testing of chemicals are widely used by the community to ensure standardization and to optimize accuracy [19, 20]. These guidelines are periodically reviewed in light of scientific progress; however, the traditional manual counting of MN is a labour-intensive process, prone to observer bias and interanalyst variability, which significantly compromises the reproducibility of results, particularly for large datasets [21, 22]. To overcome these obstacles, researchers first investigated computer-assisted methods, examples of which include threshold-based image processing and morphological filtering. For example, Rodrigues measured features (such as area, aspect ratio, and spot counts on the nuclear channel) from single-cell images of cells captured using an imaging flow cytometer to employ a gating strategy to detection phenotypes, including mono- and binucleated cells with and without MN [23]. The multiendpoint Imaging Flow Cytometer assay recently demonstrated in TK6 cells [24] could be adapted for buccal epithelial cells to extend the BMCyt assay. Following cytobrush collection, fixation, and DNA counterstaining, for example with DRAQ5, additional markers such as γH2AX for signs of DNA damage and centromere/kinetochore staining to classify clastogenic versus aneugenic MN could be incorporated. Computer-assisted image analysis would then allow automated identification of nuclei and MN, enabling higher throughput, reduced scorer bias, and mechanistic insight beyond manual scoring.

## AI-based approaches for micronucleus detection

**Figure 2:**
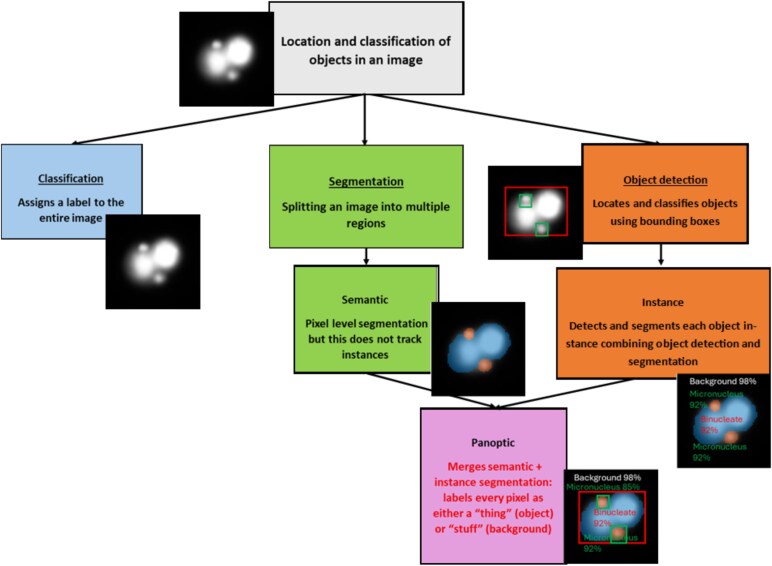
Schematic.

Although these methods increased efficiency, they faced challenges due to variations in cell morphology and staining intensity, restricting their effectiveness in complex biological samples [25]. These limitations led research groups to explore the use of machine learning and deep learning methods [26, 27]. The limitations of early computational approaches to MN detection paved the way for AI-powered techniques, particularly deep learning models, which have significantly enhanced precision and flexibility. In contrast to conventional image feature methods, AI models learn intricate patterns from large datasets, thereby increasing accuracy and consistency in MN detection [26]. Various deep learning models, especially convolutional neural networks (CNNs), have been successfully applied to automate the classification of individual images or the detection of individual objects within an image [4]. These are shown in Fig. 2.

## Classification deep learning models for micronucleus detection

Classification neural networks are designed to categorize an entire input image into one of several predefined classes. These networks typically employ an architecture where initial convolutional layers extract hierarchical features, detecting patterns, edges, and textures, while subsequent pooling layers reduce dimensionality. The extracted features are then fed into fully connected layers, which ultimately output a probability distribution indicating the likelihood of the image belonging to each class [28]. Building on their previous work detecting MN using an imaging flow cytometer [29], Rodrigues et al. used the Amnis AI imaging flow cytometry analysis software, which utilizes classification CNNs for high-accuracy scoring of imaging flow cytometry data [9]. Similarly, Wills et al. [25] used a CNN to score multiple classes of cells, including MN events, allowing for interlaboratory automation of the *in vitro* micronucleus assay. Another research effort developed software for rapid and automatic detection of MN in Giemsa-stained binucleated lymphocyte images by integrating cytokinesis-block micronucleus (CBMN) analysis with a CNN. This approach demonstrated the potential for standard automated analysis in genotoxicity testing [30].

## Object detection models for micronucleus detection

In contrast, object detection neural networks go beyond simple classification; their purpose is to both locate and classify multiple distinct objects within an image. These models achieve this by simultaneously predicting bounding boxes that delineate the position and size of each object, alongside providing a class label and confidence score for each detected instance. Architectures like YOLO (You Only Look Once) achieve this in a single forward pass, whereas two-stage detectors such as Faster R-CNN first propose regions of interest and then classify and refine those proposals, showcasing how these sophisticated networks precisely identify and contextualize specific elements within complex visual data. Faster R-CNN employs a two-stage approach, first generating region proposals and then classifying them, which, while more computationally demanding, can offer higher accuracy in object localization [31], while YOLO is renowned for its real-time processing abilities, treating detection as a single regression problem allowing for instant analysis [32]. Both models have been effectively applied to MN detection, enhancing both the accuracy and speed of identifying these biomarkers. For example, a study presented an improved YOLOv7 model that is designed for small object detection [33]. In this approach, the YOLOv7 model was optimized to deal with the distinctive characteristics of MN images, such as varying sizes and overlapping structures. The enhanced model achieved higher accuracy and faster processing times, making it suitable for high-throughput screening in genotoxicity assessments. This example illustrates the practical application of object detection algorithms in biomedical imaging, showcasing their potential to improve the efficiency and reliability of MN detection. By employing a RCNN, Panchbhai *et al.* developed a deep learning workflow to quantify MN in DNA damage studies using cultured cancer cell lines, achieving an average precision of >90% [4]. Another study proposed a computer-aided diagnosis method combining CNNs and visual attention mechanisms to recognize MN. Their model, based on a modified AlexNet architecture, incorporated attention modules to highlight regions of interest, enhancing interpretability and achieving elevated levels of accuracy in MN recognition [34].

## Segmentation models for micronucleus detection

Image segmentation is the process of dividing an image into multiple segments or regions, typically assigning a label to each pixel. All the pixels with the same label value form a mask for that particular object. These techniques can be used to extract useful information from images and aid computers to fully understand complex visual information. Thresholding and edge detection techniques can be used; however, machine learning models have been applied to good effect, often outperforming other techniques [35]. A range of machine learning models have been developed for the purposes of segmentation, and among the most popular is U-Net. U-Net is a neural network that first compresses an image to learn the broad structures, and then reconstructs it to full resolution while combining those broad features with fine details, so every pixel can be classified accurately. U-Net is a convolutional neural network designed to be successfully trained on smaller sample sizes. U-Net’s architecture enables it to classify pixels while preserving spatial details about the image. U-Net segmentation models have been effectively utilized in MN detection to achieve precise pixel-wise classification. For example, a study developed an image analysis pipeline for the automated segmentation of MN, enabling the detection and sorting of micronucleated cells. This approach facilitated the identification of cells containing MN; this is essential for understanding the consequences of micronucleation [36]. In this study, the U-Net model was trained to recognize and delineate MN within various cell types and imaging conditions. The automated segmentation achieved high sensitivity, accurately identifying MN even in challenging scenarios involving overlapping or irregularly shaped structures. This level of precision is crucial for accurate quantification in genotoxicity assays, as it ensures reliable detection and analysis of MN, thereby enhancing the assessment of DNA damage and chromosomal instability. Moreover, this approach offers a clear solution to some of the challenges presented by the traditional image-based classification, which include difficulties when dealing with rare phenotypes, complexity presented by the number of possible configurations of tri-, tetra-, or quadranucleated cells, and issues around classification imbalances. Advanced segmentation techniques deal well with these challenges by breaking the image into individual components. Rather than requiring the model to explicitly learn rare components, segmentation enables their detection as distinct instances. This helps address classification imbalance, as complex phenotypes can be reconstructed from simpler building blocks. In turn, such models tend to generalize effectively when encountering novel or previously unseen configurations [30]. The application of U-Net in this context emphasizes its suitability for biomedical imaging tasks that require detailed segmentation, contributing significantly to advancements in automated cytogenetic analysis. Another notable example of segmentation models applied to MN detection involves the development of an enhanced U-Net architecture, known as U-Net+. This model was designed to improve the segmentation of cell nuclei in microscopy images, addressing challenges such as varying nuclear sizes, shapes, and overlapping structures. The U-Net+ model incorporates modifications in the encoding branch to enhance feature extraction capabilities, leading to more accurate delineation of nuclear boundaries. This approach has demonstrated improved performance in segmenting complex nuclear images, which is crucial for accurate quantification in genotoxicity assays [37]. In this study, the U-Net+ model was trained on a diverse dataset of microscopy images, enabling it to generalize well across different cell types and imaging conditions. The enhanced architecture allowed for better handling of overlapping nuclei and varying intensities, a common challenge in microscopic image analysis. The application of U-Net+ in this context emphasizes the potential of advanced segmentation models to improve the accuracy and efficiency of automated MN detection, facilitating large-scale genotoxicity screenings and contributing to a better understanding of DNA damage and chromosomal instability.

Despite the fact that Wills *et al.* evaluated lymphocyte MN, and not buccal cells, their findings on automated scoring accuracy provide a useful benchmark for the application of BMCyt [25]. The DeepFlow system achieved an accuracy of 98% for mononucleated and an accuracy of 95% for binucleated cells, while micronucleated phenotypes were classified at 82%–85% accuracy [25]. These results compare favourably with the performance of manual scorers in buccal assays, where an interscorer variability of 15%–25% is commonly reported, particularly when identifying small or faintly stained MN [13, 38]. Manual buccal MN scoring is further complicated by the frequent presence of nuclear anomalies such as karyorrhexis, pyknosis, or binucleation, which increase subjectivity and reduce reproducibility.

Practically, both lymphocyte and buccal assays rely on the same nuclear features, identification of intact nuclei and recognition of small, rounded, nonrefractive MN within the cytoplasm, meaning that the challenges in distinguishing MN from artefacts are conceptually similar across cell types. The primary differences lie in cell morphology and staining protocols, rather than in the fundamental detection task. Against this backdrop, the reproducibility of the automated pipeline, including its strong performance on complex phenotypes of up to 96% accuracy, this suggests that DL-based object detection and segmentation approaches could substantially improve standardization in buccal MN analysis [25]. Crucially, the consistency of the automated method across laboratories highlights its potential to reduce observer bias, a longstanding limitation of traditional BMCyt assays.

Building on this, recent Deep Learning approaches extend the promise of standardization beyond feature-engineered systems such as DeepFlow. CNNs trained directly on raw image data are now able to match or outperform classical pipelines for the CBMN assay [9, 34], with strong generalization across laboratories and instruments [25]. For IFC, CNN-based scoring has been shown to reproduce expert microscopy and outperform feature-based analysis templates [9], while preserving high sensitivity for subtle, low-contrast MN, historically the main source of scorer disagreement [39].

Together with object-detection and segmentation frameworks such as Faster R-CNN and YOLO for localization and U-Net variants for pixel-level delineation, advancement in workflows report robust MN detection often >85%–90% on held-out test sets and stable performance across diverse imaging conditions [39]. In practical terms, this consistency helps overcome the interscorer variability that has long limited the BMCyt assay, making DL pipelines a strong candidate for delivering standardized and high-throughput buccal MN analysis [24]. When integrated with IFC or modern slide-scanning platforms, these approaches become even more powerful for large-scale biomonitoring [29].

## Challenges in AI-based micronucleus detection

Despite significant advancements in deep learning for MN detection, several challenges remain that obstruct widespread adoption of AI in MN detection; many of these are specific to buccal cell MN. One major limitation is data availability and annotation. Developing robust AI models requires large amounts of accurately annotated images; however, obtaining such datasets is labour-intensive and requires knowledge of the subject, which can reintroduce subjectivity and variability. For instance, when building new AI models, obtaining sufficient images of cells with MN is a significant challenge, emphasizing the importance of comprehensive image databases [40]. Buccal cells present an additional challenge, that being the lack of positive controls, in contrast to the traditional MN assays, where cultured cells can be treated with known genotoxins to induce positive responses [7]. Despite the various advantages offered by the noninvasive nature of obtaining buccal cell samples, these methods come at the cost of experimental control. Validation and interpretation of these samples are based on population analysis and comparative work, further emphasizing the need for large samples of high-quality data. Another challenge is model generalization and bias. AI models trained on specific datasets may not perform optimally across diverse imaging conditions or cell types, leading to potential biases. This issue is highlighted in discussions about biases in AI for medical imaging, where systematic errors can adversely affect patient outcomes [41]. Computational constraints also pose significant limitations. Deep learning models, especially those based on CNNs, demand substantial computational resources for both training and inference. This requirement can be restrictive for laboratories without access to high-performance computational power. The complexities inherent in medical imaging, which make it ideal for analytics algorithms, are the same intricacies that can hinder AI use [42]. Furthermore, the interpretability of AI models remains a critical issue. The ‘black box’ nature of numerous neural networks makes it challenging to understand the decision-making processes behind many predictions made by AI. This can lead to scepticism among clinicians and researchers regarding the reliability of AI-driven assessments. Efforts to enhance transparency and interpretability are essential to foster trust and facilitate integration into standard practices [43]. Addressing challenges such as these is crucial for the effective and reliable application of AI in MN detection. Ongoing research aims to develop solutions that mitigate these issues, paving the way for more robust and trustworthy AI applications in biomedical imaging.

To provide a comprehensive overview of the key challenges and potential opportunities in AI-based buccal MN detection, Table 1 summarizes information on data quality, model performance, and standardization. Problems such as class imbalance, cell variation, interpretability, and lack of widely accepted protocols are presented alongside proposed solutions, such as multimodal integration, domain adaptation, and explainable AI approaches. This table serves as a reference point to understand the barriers and strategies for growth that are influencing the future of automated genotoxicity testing.

**Table 1:** The challenges and opportunities for using AI scoring of the buccal cell MN assay.

**Challenge**	**Details**	**Opportunity**
**Data quality and annotation**	- Acquiring large, high-quality, annotated datasets of buccal cells for training deep learning models is challenging. [44] - Buccal cells vary in shape, size, and staining intensity, making high-resolution imaging complex. [45] - Manual labelling (identifying MN and other biomarkers) is time-consuming and requires expertise.	- Advanced annotation tools (e.g. active learning, semisupervised learning) can reduce manual effort and improve model generalization, even with smaller datasets.
**Cell variability**	- Buccal cells exhibit significant morphological differences due to factors like age, lifestyle, and health status. [44] - Variations in sample collection, staining methods, and imaging protocols across different studies can affect model performance. [46]	- Transfer learning and domain adaptation techniques allow models to be fine-tuned for specific datasets, improving generalization across different populations and imaging conditions. [47]
**Imbalanced data**	- MN events in buccal cells are rare compared to normal nuclei, leading to class imbalance. This imbalance can cause deep learning models to be biased toward detecting healthy cells, reducing sensitivity to MN. [48]	- Data augmentation, synthetic data generation (e.g. GANs), and specialized loss functions (e.g. focal loss) can help improve model sensitivity to rare events. [49]
**Interpretability**	- AI models, particularly CNNs, are often ‘black boxes’, making it difficult to interpret their decisions. [50] - Understanding why a model classifies a nucleus as an MN is crucial for clinical and regulatory acceptance. [51]	- Explainable AI (XAI) techniques, such as saliency maps and attention mechanisms, can provide insights into what features the model focuses on, increasing trust in AI predictions. [52]
**Standardization and validation**	- There is no universal standard for AI-based buccal cell MN detection, making cross-study comparisons difficult. [53] - Regulatory acceptance of AI-driven analysis in genotoxicity testing is still in early stages. [54]	- Developing standardized imaging protocols and validation metrics can facilitate the integration of AI models into regulatory frameworks like OECD and International Council for Harmonisation of Technical Requirements for Pharmaceuticals for Human Use (ICH) guidelines. [55]
**Automated image analysis**	- Artefacts (e.g. debris, background noise) and staining inconsistencies can affect AI performance. [56, 57] - Variability in human annotations introduces inconsistencies in training data. [58, 59]	- Establishing standardized imaging and annotation protocols will improve data quality and reduce errors in AI-based detection.
**High-throughput screening**	- Large-scale buccal MN screening is hindered by issues like false positives/negatives and inconsistencies in sample preparation. [18, 60, 61] - Differences in staining techniques affect image quality and reliability. [62]	- Data cleaning techniques, such as normalization and outlier detection, can enhance AI performance. Standardizing assay conditions can further improve consistency. [63]
**Multimodal data integration**	- Buccal MN data must be integrated with other biomarkers (e.g. oxidative stress, inflammation) for comprehensive risk assessment. [62] - Differences in data formats make integration challenging. [64]	- AI models incorporating multimodal learning (e.g. combining imaging with genomic or metabolomic data) can improve predictive accuracy for health risk assessments. [65, 66]
**Real-time monitoring and feedback**	- AI-driven real-time screening of buccal MN faces challenges like data latency, processing delays, and scalability. [67] - Large datasets require efficient filtering and processing. [68, 69]	- Edge computing and cloud-based architectures can improve real-time data analysis, making AI-powered buccal MN screening more efficient.
**Cross-population generalization**	- Genetic, lifestyle, and environmental factors cause variability in buccal MN frequency. [70–72] - AI models trained on one population may not generalize well to others. [73]	- Training models on diverse datasets and using domain adaptation techniques can enhance cross-population applicability, making AI-powered buccal MN screening more universally reliable.
**Personalized health monitoring**	- Individual differences in response to genotoxic agents make personalized assessments difficult. - AI-driven toxicity models require large, diverse datasets for accurate personalized predictions.	- AI-powered assays can potentially enable personalized buccal MN monitoring, identifying individuals at higher risk of genetic damage from environmental or lifestyle factors.

## Future directions

The integration of AI, particularly deep learning, has significantly advanced MN detection by improving accuracy, efficiency, and automation compared to traditional methods. Object detection models such as YOLO and Faster R-CNN have demonstrated the ability to identify MN with high precision, while segmentation models like U-Net have enabled more detailed morphological analysis. Despite these advancements, challenges remain, including data availability, model generalization, computational constraints, and the interpretability of AI-based decisions. Addressing these limitations will be critical for the widespread adoption of AI in genotoxicity assessment and biomedical research, particularly when dealing with buccal cell MN. As mentioned above, these cells present their own set of unique challenges, including increased variability due to hydration, oral hygiene, inflammation, and natural interpatient differences. Even within healthy individuals, cells can vary significantly in size, morphology, and staining intensity, meaning batch effects between patients or experimental runs can be particularly problematic [74]. If AI is to be used to good effect on these types of cells, generalized models are essential. Training models on masks, combined with preprocessing strategies that minimize batch effects, may help improve model robustness [74]. Transfer learning can also play a critical role: by leveraging networks pretrained on large, diverse datasets, it becomes possible to adapt existing feature representations to these smaller, domain-specific datasets, reducing the need for vast amounts of annotated data [75]. Since it is often necessary to score multiple cytogenetic endpoints, large, high-quality datasets remain crucial, but transfer learning and careful data preprocessing can substantially improve the likelihood of success [76]. Future research should focus on the development of larger, well-annotated datasets to improve model robustness across diverse imaging conditions. Additionally, explainable AI (XAI) techniques and adaptation strategies should be further explored to enhance model transparency and generalizability. Hybrid approaches that combine object detection and segmentation may also provide more comprehensive assessments of MN, improving both classification and localization accuracy. With continued innovation, AI-driven MN detection has the potential to revolutionize genotoxicity assays, contributing to more efficient and standardized biomedical diagnostics.

## Conclusion

In summary, deep learning and object classification have the potential to revolutionize the MN assay and cytome assay by enhancing the accuracy, speed, and scalability of cell analysis. The opportunities include high-throughput screening, multimodal data integration, and real-time monitoring, all of which could significantly improve genotoxicity testing and its application in regulatory and clinical settings. However, challenges such as data quality, model interpretability, and standardization remain important hurdles to overcome. Additionally, there are many challenges within AI that must also be overcome, such as data latency and alignment between species. Overcoming these challenges with the help of innovative techniques, such as transfer learning, explainable AI, and standardized validation protocols, could pave the way for deep learning’s broader adoption in cytotoxicity and genotoxicity testing.
